# Gating of miRNA movement at defined cell-cell interfaces governs their impact as positional signals

**DOI:** 10.1038/s41467-018-05571-0

**Published:** 2018-08-06

**Authors:** Damianos S. Skopelitis, Kristine Hill, Simon Klesen, Cristina F. Marco, Patrick von Born, Daniel H. Chitwood, Marja C. P. Timmermans

**Affiliations:** 1 Cold Spring Harbor Laboratory, One Bungtown Road, Cold Spring Harbor, NY 11724 USA; 2 Center for Plant Molecular Biology (ZMBP), University of Tübingen, Auf der Morgenstelle 32, 72076 Tübingen, Germany; 3 Present Address: Department Horticulture and Computational Mathematics, Science & Engineering, Michigan State University, 1066 Bogue Street, East Lansing, MI 48824 USA

## Abstract

Mobile small RNAs serve as local positional signals in development and coordinate stress responses across the plant. Despite its central importance, an understanding of how the cell-to-cell movement of small RNAs is governed is lacking. Here, we show that miRNA mobility is precisely regulated through a gating mechanism polarised at defined cell–cell interfaces. This generates directional movement between neighbouring cells that limits long-distance shoot-to-root trafficking, and underpins domain-autonomous behaviours of small RNAs within stem cell niches. We further show that the gating of miRNA mobility occurs independent of mechanisms controlling protein movement, identifying the small RNA as the mobile unit. These findings reveal gate-keepers of cell-to-cell small RNA mobility generate selectivity in long-distance signalling, and help safeguard functional domains within dynamic stem cell niches while mitigating a ‘signalling gridlock’ in contexts where developmental patterning events occur in close spatial and temporal vicinity.

## Abstract (web summary)

Movement of small RNA between cells is critical to plant development and stress responses. Here the authors uncover a gate-keeping mechanism that can restrict small RNA movement at cell-cell interfaces, providing selectivity in long-distance signalling and limiting the scope of local mobility.

## Introduction

The movement of small RNAs is fundamental to the growth and survival of plants. Small RNAs move from cell-to-cell via plasmodesmata^1^, as well as systemically through the phloem to coordinate abiotic and biotic stress responses across the plant (see refs. ^2–7^). Particularly, the spread of siRNA-mediated gene silencing is one of the main defence mechanisms against viral attack and the damaging effects of transposons (see refs. ^8–10^). Similarily, miRNAs induced in response to nutrient stress, such as phosphate, copper, or sulphur deprivation, are transported through the phloem to coordinate physiological responses between the shoot and root^2,3,11,12^.

More recently, small RNA mobility emerged as a unique and direct mechanism through which to relay positional information and drive developmental patterning^13–17^. The specification of adaxial-abaxial polarity in developing leaves relies on two opposing small RNAs, tasiARF and miR166, that generate sharp ‘on-off’ gene expression boundaries of their respective targets via an intrinsic and direct threshold-based readout of their mobility gradients^13,17,18^. miR166 also serves as a short-range positional signal in the root, where its movement from the endodermis leads to the specification of discrete cell fates in the central stele^14,15^. Further, the movement of miR394 from the epidermis of the shoot stem cell niche into the underlying two cell layers enables these cells to retain stem cell competency via down-regulation of the F-box target,  *LEAF CURLING RESPONSIVENESS* (*LCR*)^16^.

Small RNAs have properties that set them apart from other developmental signals, such as hormones, peptide ligands, and mobile transcription factors; namely, a high degree of specificity and a direct mode of action that allows for precise and rapid cell fate transitions. Small RNA regulation also confers sensitivity and robustness onto gene regulatory networks^19,20^, and the morphogen-like readout of small RNA mobility gradients yields sharply delineated domains of target gene expression^17^. These properties make mobile small RNAs particularly well suited to drive developmental change, providing a mechanism to solve the mechanistically challenging problem of generating robust and uniform developmental boundaries even under fluctuating environmental conditions.

A further conceivable advantage of employing mobile small RNAs in development may be that they represent yet another class of signals, whose movement could occur through distinct paths and be regulated by independent mechanisms. However, despite its central importance, remarkably little is known regarding the local cell to cell movement of small RNAs, except that this occurs via plasmodesmata^1^. The emphasis has been on understanding the vascular transport and reiterative spread of highly abundant and transitive siRNAs (see refs. ^6,8,9,21–23^). While these studies have been informative with respect to the propagation of RNA silencing at the whole organ or plant level, the insights gained are not informative in relation to the role of small RNAs as positional signals, whose movement occurs within defined spatial and/or temporal contexts.

Here, we show that miRNA mobility is a precisely regulated process. The directional movement of these central signalling molecules across specific cell–cell interfaces indicates the competence to move is determined at the cellular level via polarly localised determinants. These limit long-distance miRNA-mediated signalling, and the movement of miRNAs between functional domains within stem cell niches. Furthermore, we show that the mechanism regulating miRNA mobility acts independent of those controlling protein movement, identifying the small RNA as the mobile unit. Our findings reveal a gate-keeping mechanism in cell-to-cell miRNA mobility that generates selectivity in long-distance trafficking, and that helps safeguard functional domains within dynamic stem cell niches while mitigating a ‘signalling gridlock’ in contexts where developmental patterning events occur in close spatial and temporal vicinity.

## Results

### Cell-to-cell movement of miRNAs is developmentally regulated

The shape of miRNA gradients generated by movement from a defined epidermal source is consistent with the passive diffusion of small RNAs between cells^17^. This, however, does not preclude the possibility that small RNA mobility is developmentally regulated. The passive diffusion of small proteins, such as free GFP, is observed only in select developmental contexts, reflecting a spatial and temporal regulation of plasmodesmata aperture and structure (see refs. ^24,25^). Therefore, the first questions we addressed were whether miRNA mobility is developmentally regulated, and if so whether the pattern of regulation parallels that of small diffusible proteins. To this end, we took advantage of the previously described miRGFP sensor system^17^, in which the artificial miRNA miRGFP silences a ubiquitously expressed, cell autonomous, nuclear-localised GFP reporter (*p35S:3xNLS-GFP*) without triggering systemic silencing via biogenesis of secondary siRNAs (Supplementary Fig. 1a-i and Supplementary Table 1). Importantly, earlier findings indicate that inheritance of miRGFP through cell division is limited, and that the behaviour of this artificial miRNA reflects that of endogenous small RNAs, such as miR166 and the trans-acting siRNA tasiARF^17^.

To determine whether the movement of miRNAs and small diffusible proteins follows the same developmental regulation, we compared the pattern of miRGFP-directed GFP silencing to that of free GFP movement from the *ATML1, RbcS*, and *SUC2* promoters. These are active in the epidermis, mesophyll, and phloem companion cells, respectively (Supplementary Fig. 2a), and have been used extensively to study protein mobility (see refs. ^24,25^). When expressed from the *RbcS* promoter, free GFP and miRGFP show comparable non-cell autonomous effects, and are detectable in both the leaf epidermis and vasculature (Supplementary Figs. 3a–h and 4a, b). Likewise, both free GFP and miRGFP show non-cell autonomous patterns of activity when expressed in the epidermis (Supplementary Fig. 3i–p), although GFP fluorescence persists in the primary vasculature of *pATML1:miRGFP* leaves (Supplementary Fig. 3i–l). This, however, reflects an effective range rather than a movement barrier, as GFP silencing extends into the vasculature when levels of miRGFP in the epidermal source layer are inducibly increased (Supplementary Fig. 5^17^).

**Fig. 1:**
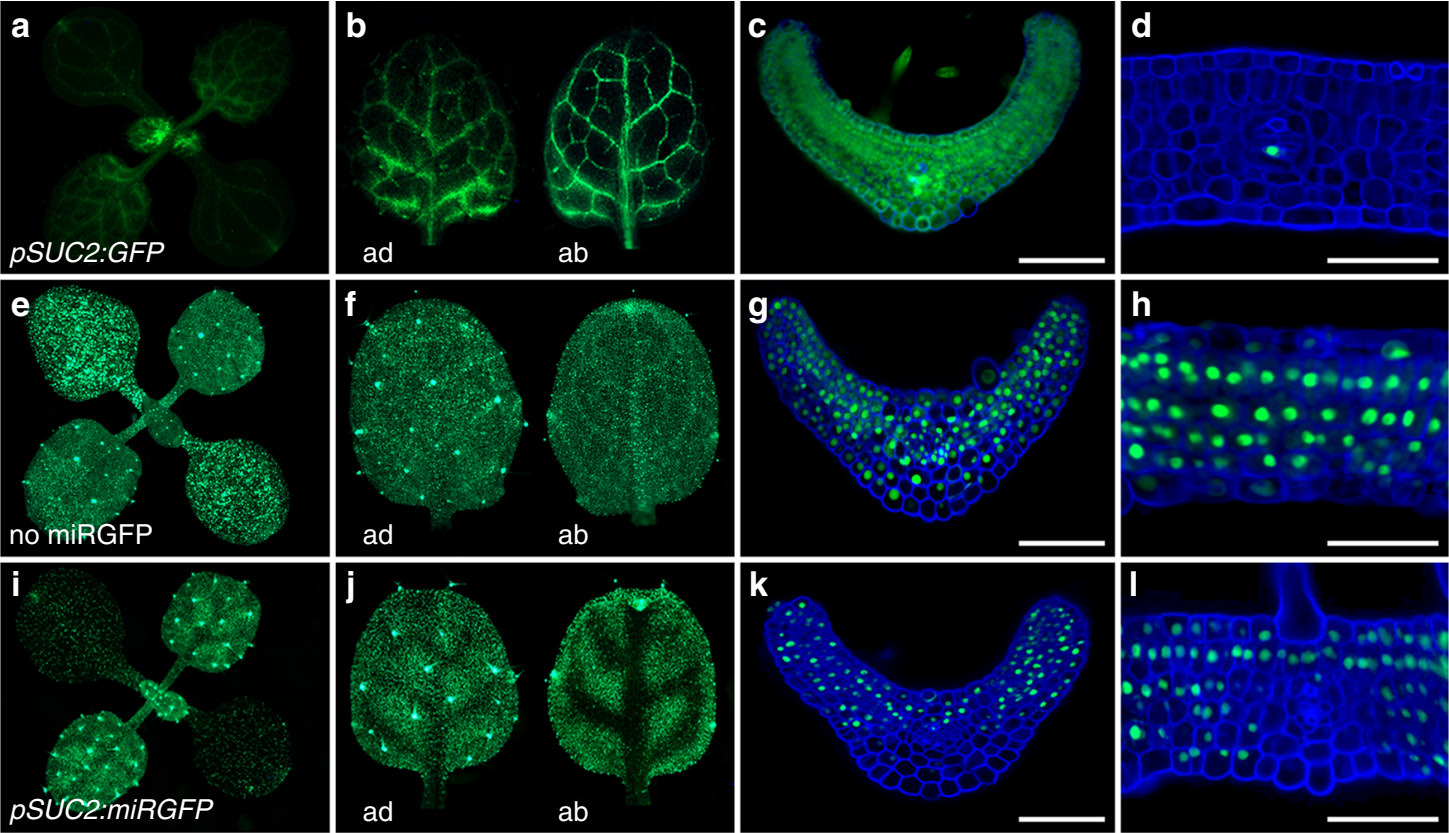
miRNA mobility is regulated independently from small protein movement. **a**–**d** Free GFP expressed in phloem companion cells (*pSUC2:GFP*) diffuses throughout **a**, **c** young sink leaves, but behaves cell autonomously in **b**, **d** mature source leaves. **e**–**h** In *p35S:3xNLS-GFP* seedlings not expressing miRGFP (no miRGFP), GFP is ubiquitously expressed. **i**–**l** miRGFP expressed in phloem companion cells (*pSUC2:miRGFP*) results in a non-cell autonomous pattern of GFP silencing that extends over 4–6 cells and appears more extensive on the abaxial (ab) side of **k** young as well as **j**, **l** mature leaves. ad, adaxial; ab, abaxial. Scale bars, 50 μm

Small proteins move freely out of phloem companion cells as well, but only in sink tissues, such as young leaves (Fig. 1a, c). In source tissues, plasmodesmatal properties change and consequently *pSUC2:GFP* lines show a cell autonomous pattern of fluorescence (Fig. 1a, b, d; see also refs. ^24,25^). Unlike free GFP, expression of miRGFP in phloem companion cells (*pSUC2:miRGFP*) results in a non-cell autonomous pattern of GFP silencing in both sink and source leaves (Fig. 1i–l). Evidence that miRGFP acts as the mobile signal comes from co-expression of the viral-suppressor protein P19. P19 sequesters 21-nt small RNA duplexes into a cell autonomous complex^9,21^ and its co-expression in phloem companion or epidermal cells eliminates the non-cell autonomous silencing effects of miRGFP (Supplementary Fig. 4c–l).

The differences in free GFP versus miRGFP mobility in source leaves are unlikely explained by differences in their molecular weight or stokes radius, even if we consider that small RNAs move in a free rather than a protein-bound form. For example, in root meristems, free GFP shows a less restrictive pattern of mobility compared to miRGFP (Supplementary Fig. 6). Fluorescence in *pSUC2:GFP* lines is phloem-restricted in the differentiation zone of the root, but GFP is efficiently off-loaded from the phloem into primary and lateral root meristems (Supplementary Fig. 6a, d, g). Conversely, in *pSUC2:miRGFP* lines, a non-cell autonomous GFP silencing pattern is only detectable in the differentiation zone (Supplementary Fig. 6). These data indicate that miRNA mobility is developmentally regulated via mechanisms distinct from those modulating basic plasmodesmatal properties, such as aperture and density, which govern the regulated symplastic diffusion of small proteins.

### miRNAs show directional mobility

**Fig. 2:**
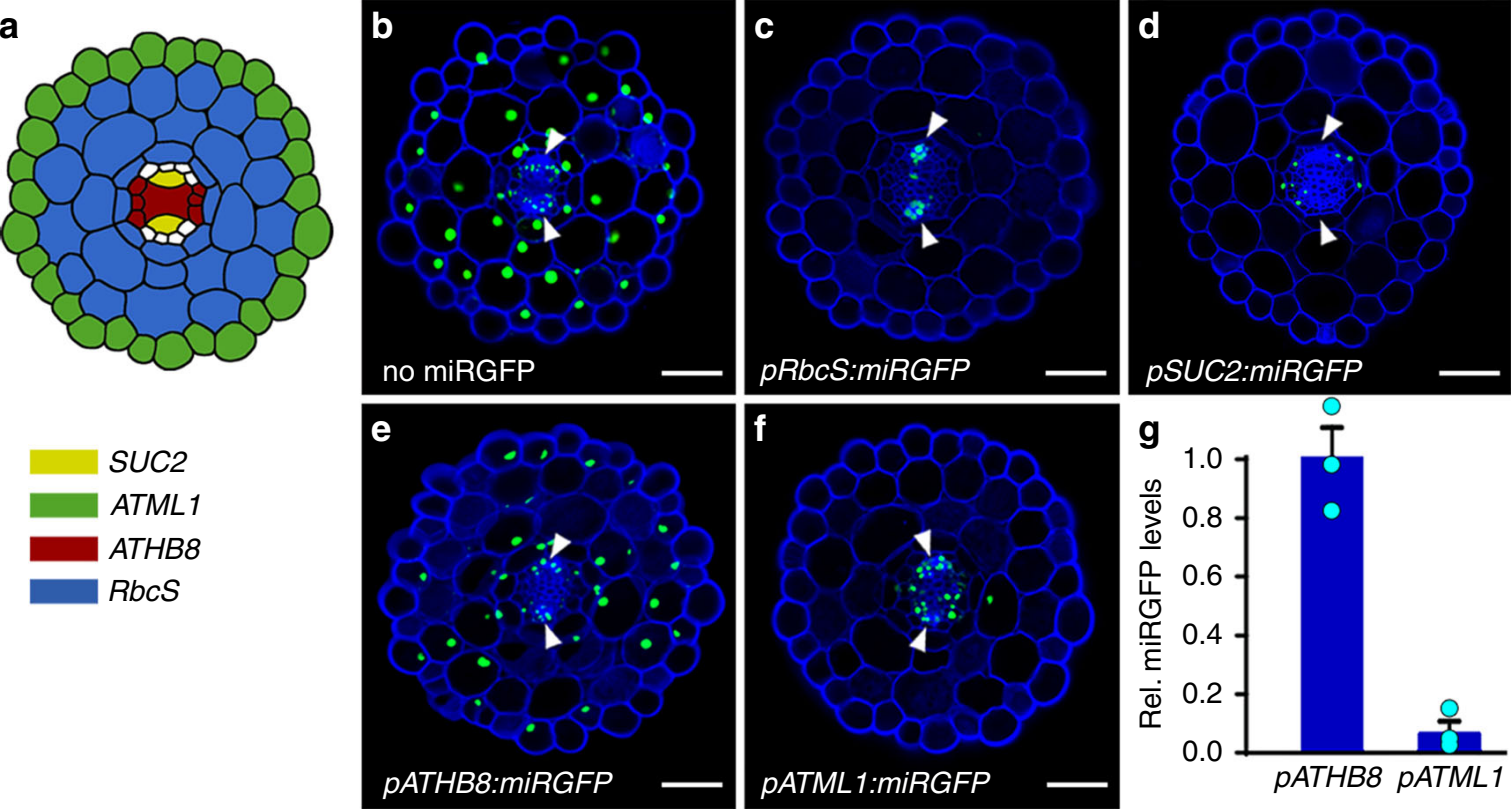
miRNAs show directional mobility. **a** Diagram illustrating the expression domains of the *SUC2* (yellow), *ATML1* (green), *ATHB8* (red), and *RbcS* (blue) promoters, as verified in Supplementary Fig. 2a. **b** Transverse section of a *p35S:3xNLS-GFP* (no miRGFP) hypocotyl shows ubiquitous GFP expression. **c**–**f** The patterns of GFP silencing in lines expressing miRGFP in **c** mesophyll (*pRbcS:miRGFP*), **d** phloem companion (*pSUC2:miRGFP*), **e** procambial (*pATHB8:miRGFP*), and **f** epidermal (*pATML1:miRGFP*) cells reveal directionality in miRGFP mobility, with miRGFP moving out but not into the phloem pole. Note, *pATHB8:miRGFP* lines show a cell autonomous pattern of GFP silencing. **g** Small RNA qRT-PCR shows that miRGFP levels are significantly (*p* < 0.001, two-sided Student’s *t*-test) higher in *pATHB8:miRGFP* vs. *pATML1:miRGFP* hypocotyls. Relative miRGFP levels (means ± SE; *n* = 3) normalised to U6 are shown. Scale bars, 50 μm. White arrowheads, phloem poles

Further evidence indicating that the movement of miRNAs is developmentally regulated comes from observations in the hypocotyl. Here, miRGFP expressed in the ground tissue (*pRbcS:miRGFP*) silences GFP in the epidermis and central stele, with the notable exception of the phloem poles (Fig. 2c and Supplementary Fig. 2a). Conversely, when expressed in the phloem companion cells (*pSUC2:miRGFP*), miRGFP silences GFP in the ground tissue and epidermis (Fig. 2d and Supplementary Fig. 2a). The conceivable caveat that miRGFP levels in *pRbcS:miRGFP* lines are below a threshold needed to clear GFP expression in cells adjacent to the source^17^, cannot explain these disparate behaviours. Small RNA deep-sequencing shows miRGFP accumulates to comparable levels in *pRbcS:miRGFP* vs. *pSUC2:miRGFP* seedlings (Supplementary Table 1), in which miRGFP levels are sufficiently high to clear GFP expression across a range of at least four cells (Fig. 2d). Also, miRGFP levels in *pRbcS:miRGFP* lines are sufficient to silence GFP in the hypocotyl procambium (Fig. 2c). Thus, whereas miRGFP is able to move out of the phloem companion cells to silence GFP in the hypocotyl ground tissue, miRGFP expressed from the *RbcS* promoter does not silence GFP in the phloem poles, indicating that miRGFP movement between endodermis and phloem is unidirectional (Fig. 2c, d).

miRNA mobility between the endodermis and vascular procambium is also unidirectional. When expressed in the procambium (*pATHB8:miRGFP*), miRGFP acts cell autonomously (Fig. 2e and Supplementary Fig. 2a). Again, levels of miRGFP cannot explain this pattern of GFP silencing. miRGFP accumulates to substantially higher levels in *pATHB8:miRGFP* than *pATML1:miRGFP* hypocotyls, which show a non-cell autonomous pattern of GFP silencing that extends across at least three cells (Fig. 2e–g and Supplementary Fig. 2a). Movement of miRGFP out of the procambium should, therefore, result in a detectable silencing effect, at least in cells immediately adjacent. Thus, the movement of miRGFP in and out of the procambium also shows directionality in a manner that is not explained by the presence of plasmodesmata or miRGFP levels at the source. Taken together, these observations reveal that miRNA mobility between neighbouring cells is regulated by a mechanism that can confer directionality across a given cell–cell interface.

### Long-distance movement of miRNAs is highly restrictive

**Fig. 3:**
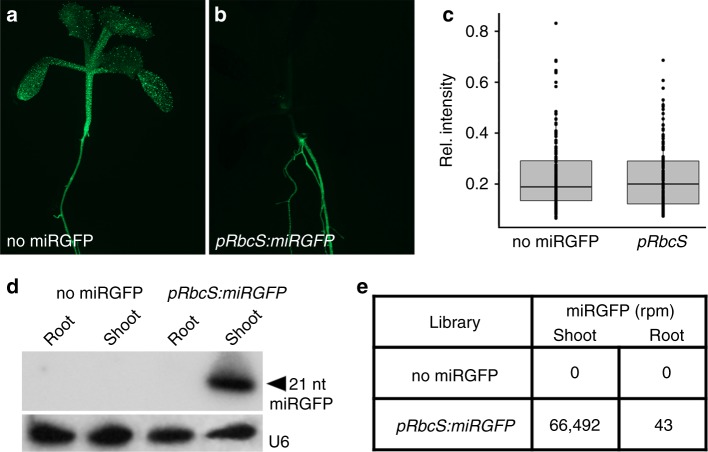
Long-distance shoot-to-root trafficking of miRNAs is limited by entry into the phloem. **a**, **b** Compared to **a** *p35S:3xNLS-GFP* (no miRGFP) seedlings, GFP silencing is apparent in the shoot but not the root of **b** *pRbcS:miRGFP* lines. **c** Quantification of the mean GFP fluorescence intensity in endodermal cells (*n* ≥ 130) normalised to fluorescence intensity in cells of the lateral root cap reveals this to not deviate significantly (*p* > 0.05, two-sided Student’s *t*-test) between *p35S:3xNLS-GFP* (no miRGFP) and *pRbcS:miRGFP* root meristems. Horizontal line, median; box, 1st to 3rd quartiles. **d** Small RNA gel blot shows miRGFP accumulates in shoots of *pRbcS:miRGFP* seedlings but is undetectable in roots. **e** Read counts for miRGFP in reads per million (rpm) normalised to the total number of mapped 19–25 nt small RNA reads in libraries constructed from shoot and root samples of *p35S:3xNLS-GFP* (no miRGFP) and *pRbcS:miRGFP* seedlings. The substantially lower levels of miRGFP in *pRbcS*:*miRGFP* roots indicate limited miRNA trafficking from shoot to root

The finding that entry into the hypocotyl phloem is restricted (Fig. 2c), has important implications for long-distance communication via miRNAs. It implies that only those miRNAs expressed in phloem companion cells are able to efficiently move long-distance from the shoot into the root. Indeed, small RNA deep-sequencing revealed that miRGFP accumulates in *pRbcS:miRGFP* seedling roots at levels three orders of magnitude below that observed in shoots (Fig. 3e). In fact, the limited loading of miRNAs into the hypocotyl phloem is likely even more extreme if we consider that miRGFP can move into the phloem in developing leaves (Supplementary Fig. 3c, d).

The minimal levels of miRGFP moving into the root are insufficient to noticeably repress GFP expression (Fig. 3a–c). Moreover, the pattern of GFP fluorescence in *pSUC2:miRGFP* roots indicates that in the absence of transitivity, miRNAs transported through the phloem fail to repress their targets in primary and lateral root meristems (Supplementary Fig. 6c, i, k). Nonetheless, a number of miRNAs are proposed to function as long-distance mobile signals to coordinate physiological responses between the shoot and root^2,3,11,12,26^. Our findings indicate that for such miRNAs to function effectively they should be produced in the phloem companion cells and trigger transitivity to propagate the silencing signal into the growing root tips. Indeed, those miRNAs reported to relay stress responses from the shoot to the root, such as miR399 in the case of phosphate deficiency, have been found to trigger transitivity^27^.

### Selective miRNA mobility in the shoot stem cell niche

As in the root, the movement of miRGFP from the central vasculature into the stem cell niche at the shoot apex is restricted. GFP fluorescence persists in the shoot apical meristem, at least prior to the floral transition, even in lines expressing miRGFP from the *ATHB8* or *RbcS* promoters, which show silencing in the vasculature and ground tissue below (Supplementary Fig. 7). Systemic transitive siRNA signals are also excluded from the shoot apex prior to flowering (see refs. ^8,9,22,28,29^). Although counterintuitive with respect to the spread of anti-viral siRNAs, a barrier at the base of the shoot apical meristem might be relevant to environmental plasticity, preventing small RNAs from establishing irreversible epigenetic change in response to transient cues^6,9,21,22^.

Nonetheless, expression of important cell fate determinants within the shoot apical meristem is regulated by miRNAs^30^, and the movement of small RNAs such as miR394 and miR166 is a key feature of their role in development^16–18^. Given the dynamic nature of the stem cell niche and the fact that cell fates are continuously defined in close spatial and temporal vicinity, miRNA mobility may need to be precisely regulated, leading us to investigate the behaviour of miRNA mobility within the shoot apical meristem.

**Fig. 4:**
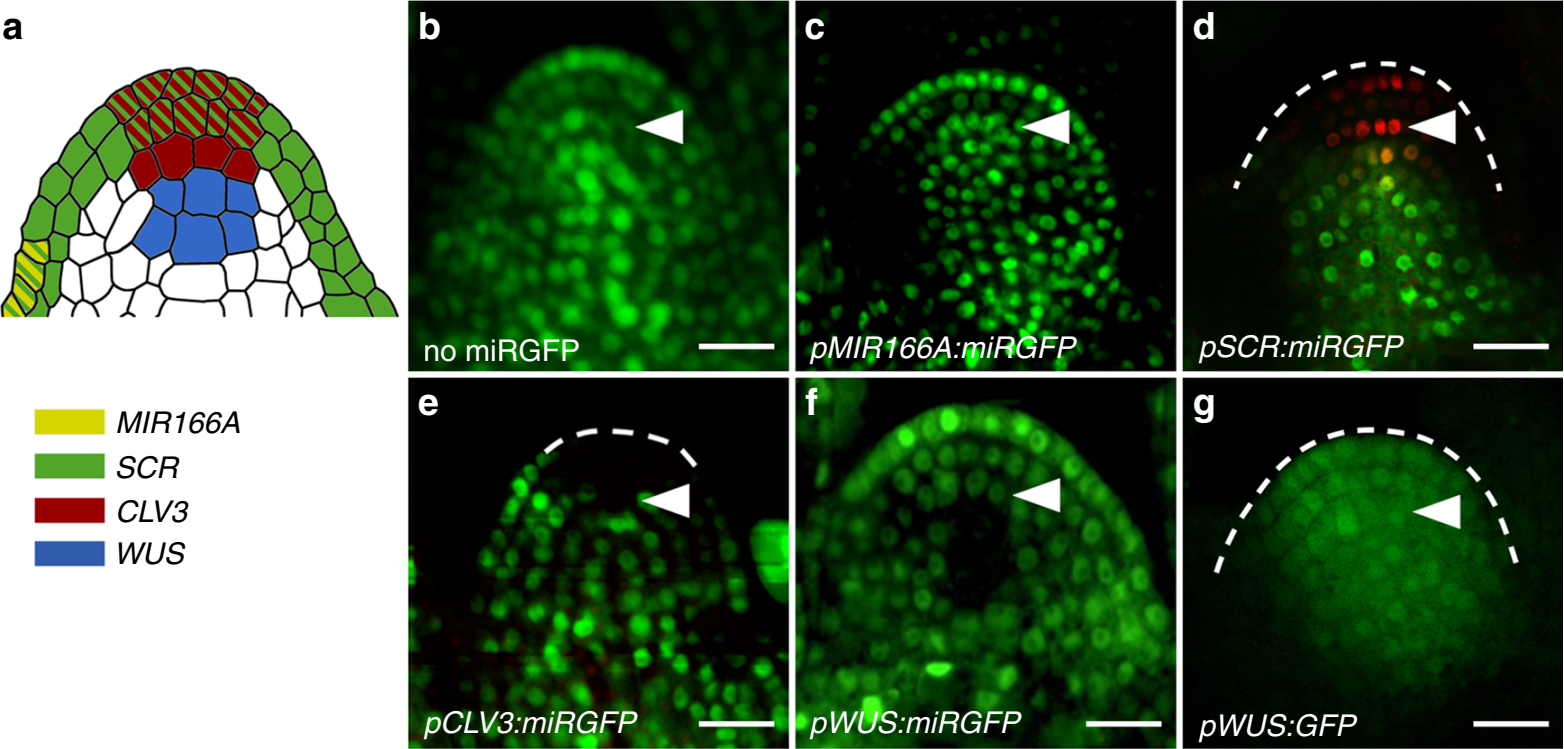
miRNAs act domain-autonomously within the shoot stem cell niche. **a** Diagram illustrating the expression domains of the *MIR166A* (yellow), *SCR* (green), *CLV3* (red), and *WUS* (blue) promoters, as verified in Supplementary Fig. 2. **b**–**g** Longitudinal sections through the shoot apical meristem of **b** a *p35S:3xNLS-GFP* (no miRGFP) seedling, and lines expressing miRGFP in the **c** abaxial epidermis of the incipient leaf (*pMIR166A:miRGFP*), **d** meristem tunica (*pSCR:miRGFP*), **e** stem cells of the central zone (*pCLV3:miRGFP*), and **f** organising centre (*pWUS:miRGFP*) show domain-autonomous patterns of miRGFP-directed GFP silencing. Cells in the central zone in **d** are marked using a *pCLV3:dsRED* reporter line. In contrast to **f** miRGFP, **g** free GFP expressed in the organising centre (*pWUS:GFP*) moves freely throughout the shoot stem cell niche. Arrowheads, third layer of the central zone. Scale bars, 10 μm

In line with the role of miR166 in specifying adaxial-abaxial polarity^17,31^, expression of miRGFP in the abaxial epidermis of incipient and developing leaf primordia (*pMIR166A:miRGFP*) results in a non-cell autonomous pattern of GFP silencing (Fig. 4a–c and Supplementary Fig. 2b). However, loss of GFP fluorescence is seen only in primordia, not elsewhere in the shoot apical meristem (Fig. 4c). Similarly, when expressed in the tunica (*pSCR:miRGFP*), miRGFP-directed silencing extends one cell layer, from the tunica into the third layer of the meristem, but GFP expression persists in the underlying organising centre and rib meristem (Fig. 4d and Supplementary Fig. 2b). These observations imply that miRNAs are able to move between cells within a given functional domain of the meristem but not between such domains.

Consistent with this idea, expression of miRGFP in the central zone *(pCLV3:miRGFP*) or organising centre (*pWUS:miRGFP*) reveals domain-autonomous patterns of GFP silencing (Fig. 4e, f and Supplementary Fig. 2b). As in the hypocotyl, these data cannot be explained by relative miRNA-to-target levels along a mobility gradient being insufficient to clear GFP expression. Deep sequencing demonstrates that miRGFP levels in *pCLV3:miRGFP* lines are almost half that of *pSUC2:miRGFP* lines, even though the *SUC2* promoter is active in many more cells throughout the seedling (Supplementary Table 1). In addition, the restrictive cell-to-cell movement of miRGFP in the shoot apical meristem is not linked to the distribution of plasmodesmata (see ref. ^29^), or the presence of pre-existing symplastic fields^32,33^, as free GFP expressed from the *WUS* promoter (*pWUS:GFP*) is able to move out of the organising centre and diffuse throughout the meristem (Fig. 4g). Instead, these data reveal the existence of additional regulatory mechanisms that spatially limit the movement of miRNAs between functional domains of the shoot stem cell niche. Importantly, given that the plasmodesmata-facilitated movement of WUS protein out of the organising centre is critical for meristem function^34,35^, the mechanism underlying the dynamic regulation of miRNA mobility in the shoot stem cell niche must also act independently of any controlling facilitated protein trafficking.

### Selective miRNA mobility is a property of stem cell niches

**Fig. 5:**
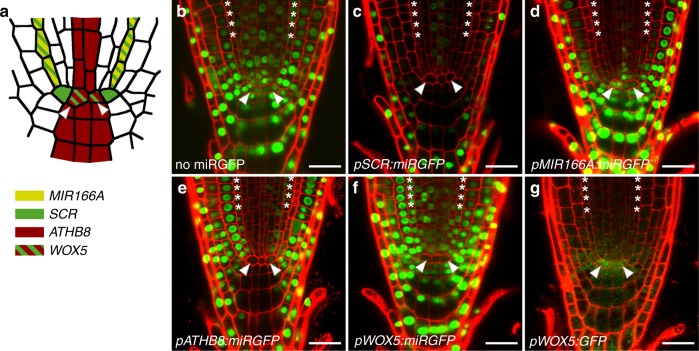
Regulated miRNA mobility within the root stem cell niche. **a** Diagram illustrating the expression domains of the *MIR166A* (yellow), *SCR* (green), *ATHB8* (red), and *WOX5* (red-green) promoters, as verified in Supplementary Fig. 2c. **b**–**g** Optical longitudinal sections through the root meristem of **b** a *p35S:3xNLS-GFP* (no miRGFP) seedling, and lines expressing miRGFP in the **c** endodermis and quiescent centre (QC) (*pSCR:miRGFP*), **d** endodermis (*pMIR166A:miRGFP*), **e** stele, QC and columella (*pATHB8:miRGFP*), and **f** QC (*pWOX5:miRGFP*), show that miRGFP acts domain-autonomously within the niche but moves between determined daughter cells of the root meristem. Note: persistence of GFP fluorescence in cells of the lateral root cap is likely explained by a low density of plasmodesmatal connections to these cells^37, 38^. In contrast to **f** miRGFP, **g** free GFP expressed in the QC (*pWOX5:GFP*) moves freely throughout the root apex. Asterisks, endodermal cells; arrowheads, QC. Scale bars, 20 μm

miRGFP when expressed in the hypocotyl procambium (*pATHB8:miRGFP;* Supplementary Fig. 2a), acts cell autonomously (Fig. 2e). The procambium comprises the vascular stem cells responsible for the continuous formation of phloem and xylem tissues. Considering the above, this observation presents the intriguing possibility that a dynamic regulation of miRNA mobility might be a general feature of stem cell niches. To address this, we analysed the pattern of miRGFP-mediated GFP silencing within the root meristem. Here, the quiescent centre is surrounded by a single layer of tissue-specific stem cells (initials) that divide asymmetrically to generate the concentrically arranged tissue files of the root, comprising the stele, cortex, endodermis, and epidermis, as well as the lateral root cap and columella^36^ (Fig. 5a).

Distinct from the binary readout of mobility-derived small RNA gradients in developing leaf primordia^17^, the movement of miR165/166 in the root generates an inversely graded pattern of HD-ZIPIII activity across the stele to pattern meta vs. proto-xylem^14,15^. The pattern of GFP silencing resulting from miRGFP expression in the root endodermis, whether from the *SCR* or *MIR166A* promoter (Supplementary Fig. 2c), is consistent with the silencing gradient of endogenous miR165/166 and extends from the endodermis into the stele and cortex (Fig. 5a–d). However, predictive of higher miRGFP source levels, a more extensive pattern of GFP silencing is seen in *pSCR:miRGFP* lines. Here, GFP silencing extends into the epidermis as well as into the columella. Importantly, the *MIR166A* promoter is not active in the stem cell niche itself (Supplementary Fig. 2c), and whereas miRGFP moves across multiple cell layers in the stele, GFP fluorescence persists in niche cells directly adjacent to the endodermis (Fig. 5d). Given that cells within the root stem cell niche are symplastically connected^37,38^, this finding implies that, as in the shoot meristem, miRGFP is unable to move from more determined cells *into* underlying stem cell initials and the quiescent centre.

Moreover, the GFP silencing pattern observed in *pATHB8:miRGFP* lines, where miRGFP is generated in the central vasculature, quiescent centre, and columella (Supplementary Fig. 2c), predicts that miRNAs are unable to move between stem cell initials or *out* of the quiescent centre. In these lines, miRGFP generates a non-cell autonomous pattern of GFP silencing in the stele, even though the *ATHB8* promoter shows a relatively low level of activity here (Fig. 5e and Supplementary Fig. 2c). In contrast, GFP fluorescence persists in the cortex/endodermis and epidermis/lateral root cap initials adjacent to the quiescent centre (Fig. 5e) where expression from the *ATHB8* promoter is comparatively strong (Supplementary Fig. 2c). Also miRGFP from the columella initials does not silence GFP in neighbouring stem cells (Fig. 5e). Mobility of miRGFP in the stele, despite limited *ATHB8* promoter activity (Supplementary Fig. 2c), argues against source levels underlying the cell autonomous behaviour of miRGFP in the niche. Instead, the GFP silencing patterns observed in the *pATHB8:miRGFP* and *pMIR166A:miRGFP* lines imply a level of regulation that permits the movement of miRNAs between more determined cells of the root meristem while limiting mobility between stem cells, and in and out of the quiescent centre. In addition, the columella presents an additional example of directional miRNA mobility, as miRGFP moves in (*pSCR:miRGFP*) but not out of the columella (*pATHB8:miRGFP*) (Fig. 5c, e).

Substantiating the finding that miRNA mobility from the quiescent centre is restricted, expression of miRGFP specifically in these cells (*pWOX5:miRGFP*) results in a domain-autonomous pattern of GFP silencing (Supplementary Fig. 2c and Fig. 5f). This restrictive miRNA activity pattern contrasts to the diffusion of small proteins, as free GFP can move both into (*pSUC2:GFP*; Supplementary Fig. 6g) and out of the quiescent centre (*pWOX5:GFP*; Fig. 5g). Furthermore, as for WUS in the SAM, symplastic movement of WOX5 protein out of the quiescent centre is essential for maintaining meristematic activity in the root tip^39^. Thus, despite their distinct organisations, miRNA mobility within the shoot, root, and vascular stem cell niches is highly regulated. Movement between the organising centre, stem cells, and more determined daughter cells within the niche is restricted, and this regulation occurs via mechanisms independent of those regulating protein trafficking, whether the diffusion of small proteins or the facilitated transport of larger transcription factors.

### miRGFP mobility reflects general behaviours of miRNAs

The pattern of miRGFP-directed GFP silencing in leaf primordia was shown to recapitulate the patterning properties of the endogenous small RNAs, miR166 and tasiARF^17^. Additionally, the highly restrictive shoot-to-root movement of miRGFP explains previously noted inefficiencies in the long-distance movement of siRNAs across graft junctions^5,26^. To further substantiate that our findings regarding miRGFP mobility reflect general behaviours of miRNAs, we developed a second synthetic sensor system based on an artificial miRNA targeting the cell autonomous GUS reporter (miRGUS). Whereas miRGFP is generated from the *MIR390A* backbone, the miRGUS design is based on the backbone of *MIR319A* (Supplementary Fig. 8a and Supplementary Table 2), which differs in its pre-miRNA structure and is processed via a different mechanism^40^.

The miRGUS system is less efficient than the miRGFP system, with miRGUS accumulating at levels sixfold lower than miRGFP expressed from the same *35S* promoter (62 rpm; Supplementary Fig. 8b vs. 391 rpm^17^). Furthermore, unlike miRGFP^17^, miRGUS biogenesis produces 20- as well as 21-nt miRNA species, thus rendering the effective levels even lower (Supplementary Fig. 8c). Nonetheless, the patterns of miRGUS-directed gene silencing observed in *pATML1:miRGUS* and *pRbcS:miRGUS* lines are comparable to those of their miRGFP counterparts (Supplementary Fig. 8d–i). Notably while GUS silencing is observed in *pRbcS:miRGUS* shoots, reporter activity persists throughout the root (Supplementary Fig. 8g). Furthermore, expression of miRGUS in the meristem tunica (*pSCR:miRGUS*) silences GUS activity in the third cell layer, but not in the underlying organising centre (Supplementary Fig. 8i), confirming the domain-autonomous behaviour of miRNAs in the shoot apical meristem (Fig. 4). Thus, mobility parameters of miRGUS mirror those of miRGFP, which in turn recapitulates the behaviour of endogenous small RNAs, including miR166 and tasiARF^14,15,17^.

Taken together, these data show that miRNA mobility is a highly regulated process that allows for directional movement in more developed tissue contexts, and domain-autonomous behaviours within stem-cell niches. The capacity for a small RNA to move is not dictated by small RNA sequence or even the pathway via which it is generated, but rather movement is spatiotemporally regulated at the cell level. This occurs independently of mechanisms governing protein movement through plasmodesmata.

## Discussion

The movement of small RNAs is fundamental to plant development, growth, and survival. Mobile small RNAs are critical for protection against the damaging effects of transposons, and in coordinating abiotic and biotic stress responses across the plant (see refs. ^9,10,13^). In addition, mobile small RNAs serve as short-range positional signals with morphogen-like activities in developmental patterning^17^. Our results show that the movement of miRNAs is a carefully regulated process, adding another level by which key responses can be controlled.

**Fig. 6:**
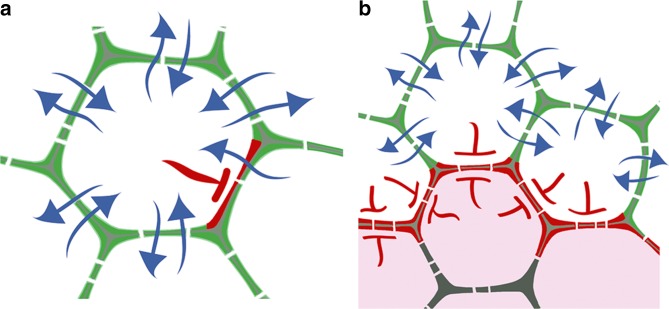
Schematic representation of polarised gate-keepers regulating the cell-to-cell movement of miRNAs. **a** Polarisation of factors that block miRNA mobility to defined cell–cell interfaces can establish unidirectional movement between neighbouring cells, or **b** when polarised to both sides of select interphases between multiple adjacent cells create domains with confined miRNA mobility. Blue arrows, miRNA movement; red line, polarised inhibition of miRNA movement; pink shading, miRNA mobility domain

The mechanisms underlying the regulated mobility of miRNAs are distinct from those controlling the facilitated transport of transcription factors such as WUS and WOX5^34,35,39^. In addition, the basic mechanisms that modulate plasmodesmata to govern the sink-source relationship and the passive diffusion of small proteins during development or in the case of stress (see refs. ^24,25,41^, cannot explain the specific instances of selective mobility described here for miRNAs. This, however, does not preclude the importance of plasmodesmata in the regulation of small RNA mobility. Instances where miRNAs are able to move from a given cell into one neighbour but not another, indicate that mobility can be regulated asymmetrically within a given cell. Examples of this include the movement of miRNAs from the ground tissue into all neighbouring cells except those of the phloem pole, and the domain-autonomous miRNA mobility observed in the stem cell niches. These reveal the presence of mobility factors that are able to polarise at defined cell–cell interfaces and function as ‘gate-keepers’ regulating the passage of miRNAs locally (Fig. 6). In this regard, it is interesting to note that many proteins, including receptors-like kinases, are preferentially located at plasmodesmata^42,43^, providing lots of scope to polarise the movement of small RNAs independently of other molecules^44^.

Regulation of miRNA mobility via polarised ‘gate-keepers’ can be envisioned irrespective of whether mobility occurs via diffusion or active transport. However, the shape of small RNA gradients observed in the leaf^17^, and their dose-dependence, favour the simple diffusion of small RNAs. In line with this notion, prior screens for factors facilitating the movement of small RNAs identified a substantial number of the known regulators of small RNA biogenesis (see refs. ^8,9,23^), but left open the question whether small RNA mobility is a regulated process. The passive diffusion of small RNAs implies that regulation occurs via ‘mobility restrictors’ preventing movement through plasmodesmata at specific cellular interfaces. Thus, perhaps analogous to the regulated movement of SHORT-ROOT and CAPRICE^45–47^, miRNA mobility is spatiotemporally regulated by mechanisms that restrict rather than facilitate movement.

Given that miRNA mobility is gated at individual cell–cell interfaces, regulated mobility cannot reflect any general sequestration mechanism. Thus, although AGO proteins act cell autonomously^9,21^, our data cannot be explained by AGO1-loading as the underpinning mechanism. Similarly, the capacity to move is not explained by miRNA overabundance, with only small RNAs exceeding the loading capacity of cell autonomous proteins, such as AGO1, moving into neighbouring cells. Levels do affect the range of mobility, but not the actual capacity to move. For example, highly expressed miRNAs in the central zone of the shoot apical meristem are domain-autonomous, whereas lowly expressed miRNAs in the epidermis silence targets across several cell layers. Considering this, and the fact that the movement of miRNAs is regulated independently from that of proteins, it is compelling to conclude that mobile small RNAs carry distinguishing marks and are themselves the mobile unit recognised by polarly localised ‘gate-keepers’.

The gating of miRNA mobility allows for selectivity in long-distance signalling. The polarised regulation of miRNA mobility at the phloem of the central vasculature generates directional cell-to-cell movement, with miRNAs moving out but not into the phloem poles (Fig. 6a). Consequently, miRNAs not produced or amplified in phloem tissues of the shoot are restricted from moving long distance into the root. Thus, the gate-keeping mechanism regulating miRNA mobility at the central vasculature creates a movement barrier that ensures some small RNA-mediated signalling responses are contained while permitting others to be propagated systemically.

Moreover, in the absence of transitivity, the root apical meristem appears protected from the activity of mobile miRNAs, as their effects are limited to the root differentiation zone. This finding is in sharp contrast to the behaviour of mobile 24-nt siRNA signals. When transported from the shoot to the root, these siRNAs, even when present at reduced levels, can direct epigenetic change in cells of the root meristem, giving rise to clonal sectors in which target genes are repressed^5,7,26^. However, while providing greater signal sensitivity, the stable epigenetic repression triggered by 24-nt siRNAs is ineffective in mediating plastic adaptive responses. Instead, miRNAs that trigger transitivity, enabling the amplification and progressive spread of silencing from a phloem source, may be particularly advantageous as long-distance messengers of environmental change. Indeed, the phloem-loaded miRNAs involved in coordinating stress responses across the plant, such as miR399 in phosphate deprivation, trigger transitivity^27,48^. This identifies miRNA precursor features and protein components required to trigger miRNA-directed transitivity as a source for selection to act upon during plant evolution.

The polarised gating mechanism also underpins a domain-autonomous behaviour of small RNAs within stem cell niches (Fig. 6b). The regulated movement of miRNAs between domains of a meristem is understandable when we consider that cell fates within stem cell niches are dynamically specified. Expression of multiple important cell fate determinants within meristems is under miRNA control (see refs. ^30,49^), but that miRNA mobility between functional domains needs stringent control is particularly well illustrated by the action of miR394. This miRNA moves from the shoot apical meristem epidermis into underlying cells where it promotes stem cell activity by repressing LCR^16^. Mechanisms that prevent the movement of miRNAs from the central zone into the organising centre below can thus help safeguard organisation in the dynamic shoot stem cell niche. Likewise, by repressing GRF transcription factor activity, miR396 ensures the properly timed transition between stem cells and transit-amplifying cells in the root stem cell niche^50^. Also, movement of miR166, while essential for the specification of adaxial-abaxial polarity in the incipient primordium^17,31^, cannot extend into the central zone where its HD-ZIPIII targets are required for stem cell activity. Mechanisms that restrict the cell-to-cell movement of miRNAs can thus help safeguard organisation in structures such as meristems (Fig. 6b), where cells in each of the domains are continuously dividing, or where miRNA levels fluctuate due to inherent noisiness in gene expression.

Still, recruiting small RNAs as a class of patterning molecules may be particularly relevant to plant stem cell niches where multiple patterning processes are occurring in close spatial and temporal vicinity. For example, within the shoot apical meristem, organ polarity is established in close proximity to signals that maintain the stem cell niche, instruct the positioning and outgrowth of the primordium, or trigger vascularisation. Careful coordination between events is thus required. The mobile signals that operate within plant stem cell niches belong to different classes of molecules, including secreted peptide ligands (e.g., CLEs), actively transported plant hormones (e.g., auxin), mobile transcription factors (e.g., WUS, WOX5), as well as mobile small RNAs (see refs. ^36,51^). The movement of these signals occurs through separate paths or, as our data indicates for plasmodesmatal trafficking, is controlled by independent regulatory mechanisms. Thus, in addition to their high specificity, favourable network properties and unique patterning outputs, an advantage of employing mobile small RNAs in development is that they represent yet another class of signalling molecules whose independent movement mitigates a ‘signalling gridlock’.

Thus, small RNAs are not simply repressors of gene expression. They are important signalling molecules whose movement is precisely regulated independently from other signals via a gating mechanism polarised at defined cell–cell interfaces. This creates selectivity in shoot-to-root phloem transport to control systemic responses at the whole plant level, and defines the scope of small RNAs as local positional cues to precisely pattern the highly dynamic plant stem cell niches.

## Methods

### Plant materials and growth conditions

All analyses were performed in the Col-0 ecotype, either wild type or *rdr6-15* (SAIL_617). The *pCLV3:dsRED* line^52^ was provided by H. Jönsson, Sainsbury Laboratory, University of Cambridge. Plants were grown at 22 °C under long-day conditions in soil or on 1% agar plates containing 1x Murashige and Skoog (MS) medium (Sigma-Aldrich) supplemented with 0 or 1% sucrose and appropriate antibiotics. β-Estradiol inductions were performed by germinating seedlings on the above media then transferring daily to 0.7% agar MS plates supplemented with fresh 20 μM β-estradiol for the indicated incubation times. Analyses on roots were performed 7–8 days after germination, and all other analyses were performed on 10–12 day-old seedlings.

### Generation of constructs and transgenic plants

The following promoter fragments were used in this study: 0.7 kb *2* × *35S*, 2.2 kb *ATML1*, 1.8 kb *RbcS*, 2.5 kb *MIR166A*, 2.3 kb *SUC2*, 2.2 kb *ATHB8*, 2.5 kb *SCR*, 2.2 kb *WUS*, 4.2 kb *CLV3* and 4.2 kb *WOX5*. Promoter fragments were amplified using PCR primers with appropriate attB1-attB2 sites (Supplementary Table 3) and introduced into pDONR207 (Invitrogen). Transcriptional GUS reporter fusions were generated by cloning these promoters upstream of the *uidA* gene in the pGreen II 0029 binary vector using Gateway technology. Transcriptional GFP reporter constructs were generated similarly by introducing the *ATML1*, *RbcS*, *SUC2*, and *WUS* promoter fragments upstream of *GFP6-6xHIS* in pMDC107. The *pWOX5:GFP* construct was generated by Gibson assembly (NEB) introducing the mGFP6 coding sequence downstream of the *WOX5* promoter in the pK7WG plasmid backbone.

The *p35S:3xNLS-GFP* reporter line and the *miRGFP* precursor have been described previously^17^. The β-estradiol inducible *pATML1»miRGFP* construct was created by inserting the miRGFP precursor into pMDC7 using Gateway technology. The G10-90 promoter of this vector was subsequently replaced by a Gateway cassette via Gibson assembly (NEB). To create the final *pATML1*»*miRGFP* construct, the 2.1 kb *ATML1* promoter was introduced upstream of the miRGFP precursor using Gateway technology. The miRGUS sequence was introduced into a 404 bp *MIR319A* precursor fragment using overlapping PCR^53^. This *MIR319A*-based *miRGUS* precursor fused to the NOS terminator was custom synthesised and cloned downstream of the Gateway cassette in the pGreen II 0029 binary vector using *Spe*I/*Sac*I restriction sites. Using Gateway technology, the *2* × *35S*, *ATML1*, *RbcS*, and *SCR* promoter fragments were introduced upstream of *miRGUS* to create *p35S:miRGUS*, *pATML1:miRGUS*, *pRbcS:miRGUS*, and *pSCR:miRGUS*, respectively. A Gateway-based p19 expression vector, with the 578 bp coding sequence of the viral-suppressor protein p19 fused to HA inserted downstream of the Gateway cassette in pGreen II 0029, was assembled by Gibson assembly (NEB). Using Gateway technology, the *ATML1* and *SUC2* promoter fragments were subsequently introduced upstream of *p19-HA* to create *pATML1:p19-HA* and *pSUC2:p19-HA*, respectively.

The *p35S:3xNLS-GFP* and *p35S:GUS* reporter lines were generated in the *rdr6-15* background and subsequently transformed with *miRGFP* and *miRGUS* precursor constructs, respectively. Multiple independent transformants per construct (*n* of between 10 and 20) were observed, of which at least four independent lines with representative behaviour were analysed in greater detail. Select *pATML1-*, *pRbcS-*, *pSUC2-*, *pSCR-*, and *pWOX5-miRGFP* lines were crossed onto Col-0 to confirm the silencing patterns also in this wild-type background. P19 constructs were initially transformed into Col-0 and subsequently introduced into *pSUC2:miRGFP* and *pATML1:miRGFP* lines via crossing. The GUS and GFP promoter fusions were also generated in the *rdr6-15* background. Artificial miRNA sequences are listed in Supplementary Table 2, and cloning primers are listed in Supplementary Table 3.

### Confocal microscopy

Ten-day-old seedlings were fixed in 4% paraformaldehyde (PFA) dissolved in 1x PBS supplemented with 0.01% Tween 20 (Sigma-Aldrich) under vacuum (33 mBar) for 45 min. Fixed tissues were washed 3 times (10 min per wash) in 1× PBS and embedded in 8% Low Melting Agarose (Invitrogen)^17^. Sections were obtained using a VT1000S vibratome (Leica). For imaging of leaf primordia and hypocotyls, 100 μm sections were acquired while, for shoot apices, the thickness was set to 50 μm. Tissue sections were stained with 0.01% Fluorescent Brightener 28 (FB) in 1x PBS (Sigma-Aldrich) for 20 min, then washed three times (10 min per wash) in 1x PBS. Imaging of tissue sections was performed using an inverted laser-scanning confocal microscope (Zeiss LSM 780). Excitation for FB, GFP and Chlorophyll was at 405, 488, and 633 nm, respectively, using 2% laser power. Image acquisition was at 410–475 nm, 491–597 nm, and 638–721 nm, respectively. For root imaging, 7-day-old roots were incubated in 10 μg/mL Propidium Iodide (PI) (Sigma-Aldrich) in water for 20 min at room temperature, and washed three times in water before imaging. Excitation of PI was at 561 nm and images were collected at 566–719 nm. Root imaging was performed using a Leica (SP8) laser-scanning confocal microscope.

For quantification of nuclear-localised GFP signal, 28.5 μm z-stacks were imaged in 0.45 μm intervals with an effective pixel dwell time of 1 μs at 1044 × 1044-pixel frame resolution. All images were collected using a bit depth of 16 bits. Nuclear-localised GFP signal in the endodermal layer of root meristems was quantified using the surfaces module of Imaris v. 8.0.2 (Bitplane). The stacks were processed using a 3 µm diameter background subtraction, with a minimum absolute intensity threshold of 75.3, and a seed detection diameter of 2–3 μm in the x-level. Z-stacks were manually processed and mean GFP intensities measured from the first eight endodermal nuclei on either side of the quiescent centre in 8–12 roots per line. GFP intensities were normalised to the mean signal intensity in lateral root cap nuclei. Values (means ± SE) were plotted and statistical significance calculated using Student’s *t*-test.

### Histology and microscopy

For GUS analyses, seedlings were harvested into ice-cold acetone and prefixed for 20 min at room temperature. Seedlings were washed with 100 mM sodium phosphate (pH 7.0), 10 mM EDTA, 0.1% Triton-X buffer with the indicated concentrations of ferrocyanide and ferricyanide, and allowed to sit on ice for 5 min. The same buffer supplemented with 0.05% X-Gluc was then added and the seedlings vacuum-infiltrated for 30 min at 600 mm Hg. The following concentrations of supplemented ferro/ferricyanide were used: 2 mM for *pATML1:GUS*, *pSUC2:GUS*, *pATHB8:GUS*, and *pWUS:GUS*, 6 mM for *pRbcS:GUS, pSCR:GUS*, and *pMIR166A:GUS*, and 10 mM for *pCLV3:GUS*. For GUS staining in roots, 10 mM supplemented ferro/ferricyanide was used for *pSCR:GUS* and *pWOX5:GUS* and 4 mM for *pATHB8:GUS* and *pMIR166A:GUS*. Seedlings were incubated at 37 °C as needed, and subsequently dehydrated to 50% ethanol, fixed in FAA (50% ethanol, 5% formaldehyde, 10% acetic acid), and embedded and sectioned^13^. Clearing of stained roots was performed overnight using 15 M chloral hydrate in 30% glycerol. Imaging of tissue sections and whole-mount GUS-stained seedlings was performed using DIC on a Zeiss Axiophot. Whole-mount fluorescence imaging was performed on a SMZ1500 dissecting microscope (Nikon), equipped with a P-FLA2 epi-fluorescence attachment.

Small RNA in situ hybridisations^54^ were performed on 10-day-old seedlings. Tissue sections from seedlings fixed in 4% PFA in 1x PBS, were treated with 0.125 mg/mL Protease for 30 min at 37 °C prior to hybridisation. Treated sections were hybridised overnight at 50 °C with double-digoxigenin labelled asmiRGFP LNA probe (Exiqon) at a concentration of 50 nM. Slides were washed twice in 0.2× SCC for 1 h each at 50 °C. Hybridisation signal was detected by immunohistochemistry using anti-DIG-AP Fab fragments from sheep (Roche) at 0.6 U/mL in 1x TBS with 1% BSA and 0.3% Triton-X-100 at room temperature for 2–4 h and visualised using NBT/BCIP mix (Roche). The asmiRGFP probe sequence is listed in Supplementary Table 2.

### Small RNA analysis

Total RNA was isolated from 10-day-old seedlings using TRIzol reagent (Invitrogen). For analysis of small RNA levels by northern blotting, 20 μg total RNA was resolved on a 17% polyacrylamide gel containing 7 M urea. Samples were transferred to Hybond N + membrane (Sigma-Aldrich), crosslinked using a Stratalinker UV crosslinker model 1800 (Stratagene), and hybridised using ^32^P end-labelled asmiRGFP probe^55^. U6 was used as loading control. Original gel images are provided in the Supplementary Information (Supplementary Fig. 9). For small RNA quantitative reverse transcription PCR (qRT-PCR) analysis, RNA was isolated from a pool of dissected 10-day-old hypocotyls using the ISOLATE II Plant miRNA Kit (Bioline) and 350 ng used for cDNA synthesis using the iScript™ cDNA Synthesis Kit. qRT-PCR was performed using multiplex primers for miRGFP and U6. Small RNA levels normalised to U6 were calculated based on three technical and three biological replicates using the ΔΔCT method and significance was tested using the two-sided Student’s *t*-test.

Small RNA libraries were constructed from 10-day-old seedlings, or when indicated from separate shoot and root samples, using the TruSeq Small RNA sample preparation kit (Illumina). Libraries were quantified with the KAPA Illumina Library Quantification Kit (Kapabiosystems), and sequenced on the Illumina HiSeq 2000 platform. Reads were trimmed using the FASTX-Toolkit ([http://hannonlab.cshl.edu/fastx_toolkit/]), and trimmed reads 19- to 25-nt in length aligned to the *Arabidopsis* reference genome (TAIR10) with GFP and GUS target sequences added using the Burrows-Wheeler Aligner^56^. For analysis of miRGFP levels, a single mismatch was allowed in the alignments to accommodate for the mismatch at position 20 of miRGFP relative to GFP. Similarly, for miRGUS, three mismatches were allowed in the alignments. Reads matching known structural RNAs (rRNAs, tRNAs, sn-RNAs, and sno-RNAs) from Rfam 10.0 were removed from further analysis. For comparison of small RNA levels across samples, read counts were normalised per million mapped reads (reads per million) using SAM Tools^56^.

### Data availability

All high-throughput sequencing data, both raw and processed files, are available through NCBI’s Gene Expression Omnibus under accession number GSE102236. The authors declare that all other data supporting the findings of this study are available within the manuscript and its supplementary files or are available from the corresponding author upon request.

## Electronic supplementary material

Supplementary Information

## References

[1] Vatén A (2011). Callose biosynthesis regulates symplastic trafficking during root development. Dev. Cell..

[2] Buhtz A, Springer F, Chappell L, Baulcombe DC, Kehr J (2008). Identification and characterization of small RNAs from the phloem of *Brassica napus*. Plant J..

[3] Lin SI (2008). Regulatory network of microRNA399 and PHO2 by systemic signaling. Plant Physiol..

[4] Ruiz-Ferrer V, Voinnet O (2009). Roles of plant small RNAs in biotic stress responses. Annu. Rev. Plant. Biol..

[5] Molnar A (2010). Small silencing RNAs in plants are mobile and direct epigenetic modification in recipient cells. Science.

[6] Zhang W (2014). Graft-transmissible movement of inverted-repeat-induced siRNA signals into flowers. Plant J..

[7] Lewsey MG (2016). Mobile small RNAs regulate genome-wide DNA methylation. Proc. Natl Acad. Sci. U.S.A..

[8] Brosnan CA, Voinnet O (2011). Cell-to-cell and long-distance siRNA movement in plants: mechanisms and biological implications. Curr. Opin. Plant Biol..

[9] Melnyk CW, Molnar A, Baulcombe DC (2011). Intercellular and systemic movement of RNA silencing signals. EMBO J..

[10] Borges F, Martienssen RA (2015). The expanding world of small RNAs in plants. Nat. Rev. Mol. Cell Biol..

[11] Yoo BC (2004). A systemic small RNA signaling system in plants. Plant Cell.

[12] Pant BD, Buhtz A, Kehr J, Scheible WR (2008). MicroRNA399 is a long-distance signal for the regulation of plant phosphate homeostasis. Plant J..

[13] Chitwood DH (2009). Pattern formation via small RNA mobility. Genes Dev..

[14] Carlsbecker A (2010). Cell signalling by microRNA165/6 directs gene dose-dependent root cell fate. Nature.

[15] Miyashima S, Koi S, Hashimoto T, Nakajima K (2011). Non-cell-autonomous microRNA165 acts in a dose-dependent manner to regulate multiple differentiation status in the Arabidopsis root. Development.

[16] Knauer S (2013). A protodermal miR394 signal defines a region of stem cell competence in the Arabidopsis shoot meristem. Dev. Cell..

[17] Skopelitis DS, Benkovics AH, Husbands AY, Timmermans MCP (2017). Boundary formation through a direct threshold-based readout of mobile small RNA gradients. Dev. Cell.

[18] Juarez MT, Kui JS, Thomas J, Heller BA, Timmermans MCP (2004). microRNA-mediated repression of *rolled leaf1* specifies maize leaf polarity. Nature.

[19] Schmiedel JM (2015). MicroRNA control of protein expression noise. Science.

[20] Plavskin Y (2016). Ancient trans-acting siRNAs confer robustness and sensitivity onto the auxin response. Dev. Cell.

[21] Chitwood DH, Timmermans MC (2010). Small RNAs are on the move. Nature.

[22] Liang D, White RG, Waterhouse PM (2012). Gene silencing in Arabidopsis spreads from the root to the shoot, through a gating barrier, by template-dependent, nonvascular, cell-to-cell movement. Plant Physiol..

[23] Taochy C (2017). A genetic screen for impaired systemic RNAi highlights the crucial role of DICER-LIKE 2. Plant Physiol..

[24] Burch-Smith TM, Zambryski PC (2012). Plasmodesmata paradigm shift: regulation from without versus within. Annu. Rev. Plant Biol..

[25] Tilsner J, Nicolas W, Rosado A, Bayer EM (2016). Staying tight: Plasmodesmal membrane contact sites and the control of cell-to-cell connectivity in plants. Annu. Rev. Plant Biol..

[26] Melnyk CW, Molnar A, Bassett A, Baulcombe DC (2011). Mobile 24 nt small RNAs direct transcriptional gene silencing in the root meristems of *Arabidopsis thaliana*. Curr. Biol..

[27] Manavella PA, Koenig D, Weigel D (2012). Plant secondary siRNA production determined by microRNA-duplex structure. Proc. Natl. Acad. Sci. U.S.A..

[28] Schwach F, Vaistij FE, Jones L, Baulcombe DC (2005). An RNA-dependent RNA polymerase prevents meristem invasion by Potato Virus X and is required for the activity but not the production of a systemic silencing signal. Plant Physiol..

[29] Kitagawa M, Jackson D (2017). Plasmodesmata-mediated cell-to-cell communication in the shoot apical meristem: How stem cells talk. Plants.

[30] Fouracre JP, Poethig RS (2016). The role of small RNAs in vegetative shoot development. Curr. Opin. Plant Biol..

[31] Husbands AY, Benkovics AH, Nogueira FT, Lodha M, Timmermans MCP (2015). The ASYMMETRIC LEAVES complex employs multiple modes of regulation to affect adaxial-abaxial patterning and leaf complexity. Plant Cell.

[32] Rinne PL, van der Schoot C (1998). Symplasmic fields in the tunica of the shoot apical meristem coordinate morphogenetic events. Development.

[33] Gisel A, Barella S, Hempel FD, Zambryski PC (1999). Temporal and spatial regulation of symplastic trafficking during development in *Arabidopsis thaliana* apices. Development.

[34] Yadav RK (2011). WUSCHEL protein movement mediates stem cell homeostasis in the *Arabidopsis* shoot apex. Genes Dev..

[35] Daum G, Medzihradszky A, Suzaki T, Lohmann JU (2014). A mechanistic framework for noncell autonomous stem cell induction in *Arabidopsis*. Proc. Natl Acad. Sci. U.S.A..

[36] Sparks E, Wachsman G, Benfey PN (2013). Spatiotemporal signalling in plant development. Nat. Rev. Genet..

[37] Zhu T, Lucas WJ, Rost TL (1998). Directional cell-to-cell communication in the *Arabidopsis* root apical meristem I. An ultrastructural and functional analysis. Protoplasma.

[38] Zhu T, O’Quinn RL, Lucas WJ, Rost TL (1998). Directional cell-to-cell communication in the *Arabidopsis* root apical meristem II. Dynamics of plasmodesmatal formation. Protoplasma.

[39] Pi L (2015). Organizer-derived WOX5 signal maintains root columella stem cells through chromatin-mediated repression of CDF4 expression. Dev. Cell.

[40] Bologna NG, Mateos JL, Bresso EG, Palatnik JF (2009). A loop-to-base processing mechanism underlies the biogenesis of plant microRNAs miR319 and miR159. EMBO J..

[41] Han X, Kim JY (2016). Integrating hormone- and micromolecule-mediated signaling with plasmodesmal communication. Mol. Plant.

[42] Fernandez-Calvino L (2011). *Arabidopsis* plasmodesmal proteome. PLoS ONE.

[43] Stahl Y, Faulkner C (2016). Receptor complex mediated regulation of symplastic traffic. Trends Plant Sci..

[44] Rosas-Diaz T (2018). A virus-targeted plant receptor-like kinase promotes cell-to-cell spread of RNAi. Proc. Natl Acad. Sci. U.S.A..

[45] Sena G, Jung JW, Benfey PN (2004). A broad competence to respond to SHORT ROOT revealed by tissue-specific ectopic expression. Development.

[46] Kurata T (2005). Cell-to-cell movement of the CAPRICE protein in *Arabidopsis* root epidermal cell differentiation. Development.

[47] Wu S, Gallagher KL (2014). The movement of the non-cell-autonomous transcription factor, SHORT-ROOT relies on the endomembrane system. Plant J..

[48] Chen HM (2010). 22-Nucleotide RNAs trigger secondary siRNA biogenesis in plants. Proc. Natl Acad. Sci. U.S.A..

[49] Couzigou JM, Combier JP (2016). Plant microRNAs: key regulators of root architecture and biotic interactions. New Phytol..

[50] Rodriguez RE (2015). MicroRNA miR396 regulates the switch between stem cells and transit-amplifying cells in *Arabidopsis* roots. Plant Cell.

[51] Benkovics AH, Timmermans MC (2014). Developmental patterning by gradients of mobile small RNAs. Curr. Opin. Genet. Dev..

[52] Willis L (2016). Cell size and growth regulation in the *Arabidopsis thaliana* apical stem cell niche. Proc. Natl Acad. Sci. U.S.A..

[53] Schwab R, Ossowski S, Riester M, Warthmann N, Weigel D (2006). Highly specific gene silencing by artificial microRNAs in *Arabidopsis*. Plant Cell.

[54] Javelle M, Timmermans MC (2012). In situ localization of small RNAs in plants by using LNA probes. Nat. Prot..

[55] Petsch K (2015). Novel DICER-LIKE1 siRNAs bypass the requirement for DICER-LIKE4 in Maize development. Plant Cell.

[56] Li H, Durbin R (2009). Fast and accurate short read alignment with Burrows-Wheeler transform. Bioinformatics.

