# Supplementary material for: Gating of miRNA movement at defined cell-cell interfaces governs their impact as positional signals
Source: Nat Commun. 2018 Aug 6;9:3107. doi: 10.1038/s41467-018-05571-0 (PMC6079027; doi:10.1038/s41467-018-05571-0)
Supplement: Supplementary file 1 — Supplementary Information [file 41467_2018_5571_MOESM1_ESM.pdf]

Gating of miRNA Movement at Defined Cell-Cell Interfaces Governs Their Impact as Positional Signals

Skopelitis, Hill et al.

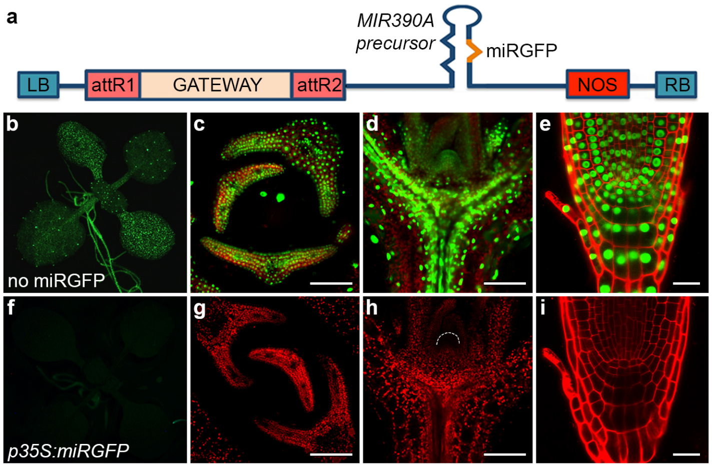

Supplementary Figure 1. miRGFP efficiently silences GFP expression. **(a)** Schematic of the Gateway-based miRGFP precursor construct. **(b-i)** GFP fluorescence in **(b-e)** *p35S:3xNLS-GFP* (no miRGFP) seedlings is completely eliminated in **(f-i)** such seedlings ubiquitously expressing miRGFP (*p35S:miRGFP*). **(b, f)** 10-day old seedlings, **(c-e, g-i)** confocal images of tissue sections from **(c, g)** young leaf primordia, **(d, h)** shoot apices and **(e, i)** root tips. Scale bars, **(c, d, g, h)** 100 µm and **(e, i)** 20 µm.

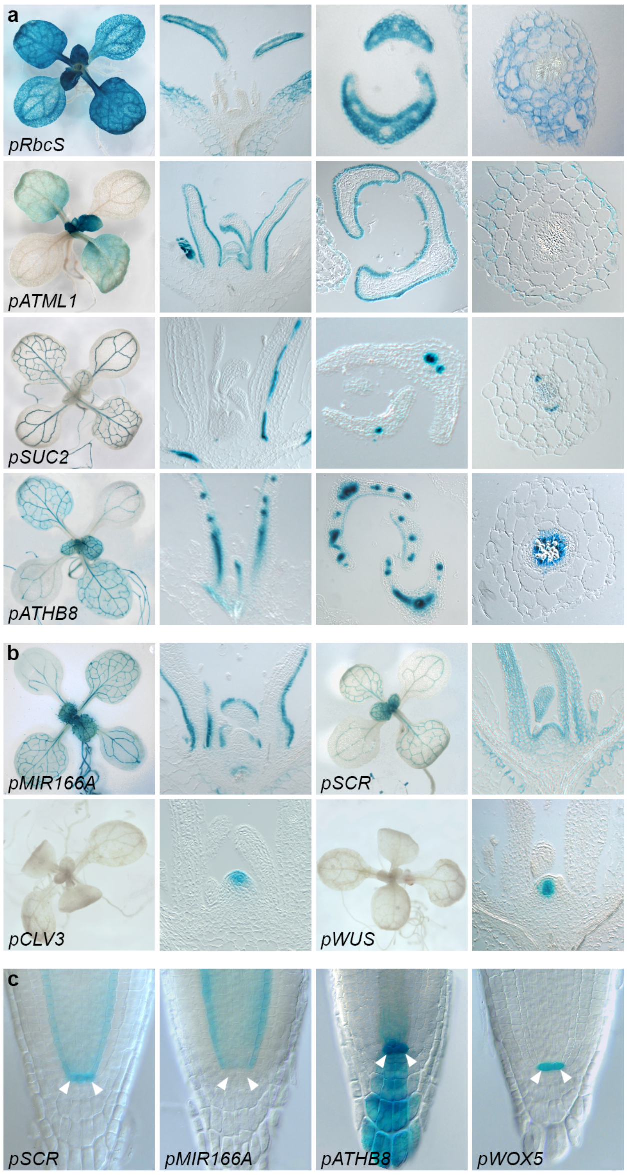

Supplementary Figure 2. All promoter fragments used in this study yield the expected spatiotemporal patterns of expression. **(a)** Transcriptional GUS reporter fusions reveal the established tissue specific patterns of activity (left to right) in 10-day old seedlings, longitudinal apex sections, transverse leaf primordia sections, and transverse hypocotyl sections for the selected *RbcS, ATML1, SUC2,* and *ATHB8* promoter fragments. GUS reporter activity in *pRbcS:GUS, pATML1:GUS, pSUC2:GUS* and *pATHB8:GUS* seedlings is limited to the ground tissue, epidermis, phloem companion cells and procambium, respectively. **(b)** Patterns of GUS reporter activity in *pMIR166A:GUS, pSCR:GUS, pCLV3:GUS* and *pWUS:GUS* 10-day old seedlings (left) and longitudinal apex sections (right) confirms these promoters are active in the SAM in the abaxial epidermis of the incipient leaf, the tunica (L1 and L2), central zone and organizing centre, respectively. **(c)** Whole mount images of the of the root apical meristem of *pSCR:GUS, pMIR166A:GUS, pATHB8:GUS* and *pWOX5:GUS* seedlings reveal the expected patterns of GUS reporter

activity in the endodermis and QC (*SCR*), the endodermis (*MIR166A*), the vasculature, QC and columella (*ATHB8*), and the QC (*WOX5*).

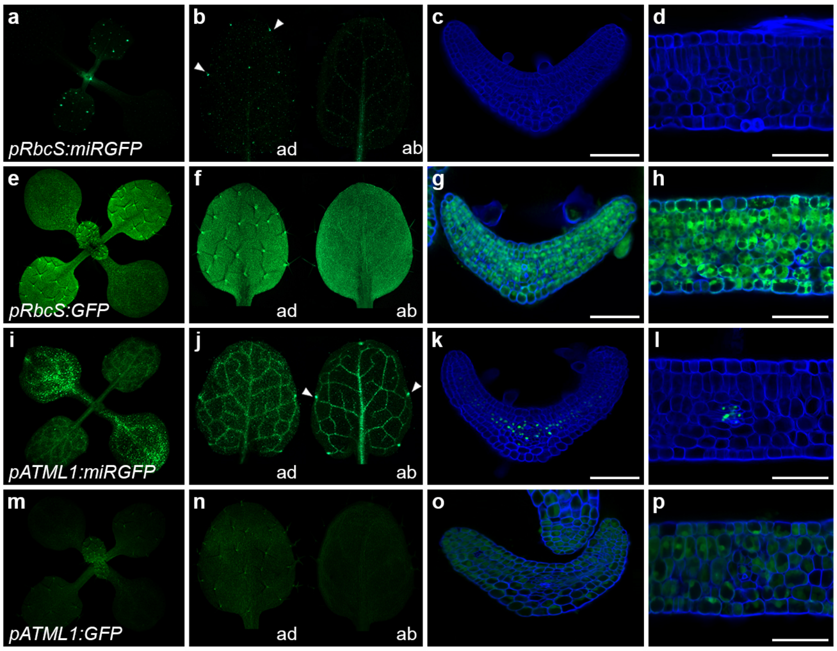

Supplementary Figure 3. miRNA mobility is regulated independently from small protein movement. **(a-h)** Imaging of **(a, e)** whole seedlings, **(b, f)** individual leaves, **(c, g)** sections of leaf primordia and **(d, h)** sections of expanded leaves reveals that miRGFP and free GFP show comparable non-cell autonomous effects when expressed in mesophyll, with **(a-d)** miRGFP-mediated GFP silencing and **(e-h)** free GFP diffusion detectable in both the epidermis and vasculature in *pRbcS:miRGFP* and *pRbcS:GFP* lines, respectively. **(i-p)** Likewise, imaging of **(i, m)** whole seedlings, **(j, n)** individual leaves, **(k, o)** sections of leaf primordia and **(l, p)** sections of expanded leaves shows the non-cell autonomous spread of **(i-l)** miRGFP-directed GFP silencing and **(m-p)** free GFP diffusion from the epidermis in *pATML1:miRGFP* and *pATML1:GFP* lines, respectively. Note, GFP fluorescence persists in the symplastically isolated trichomes and hydathodes (arrow heads), consistent with miRGFP moving through plasmodesmata. Scale bars, 50 μm.

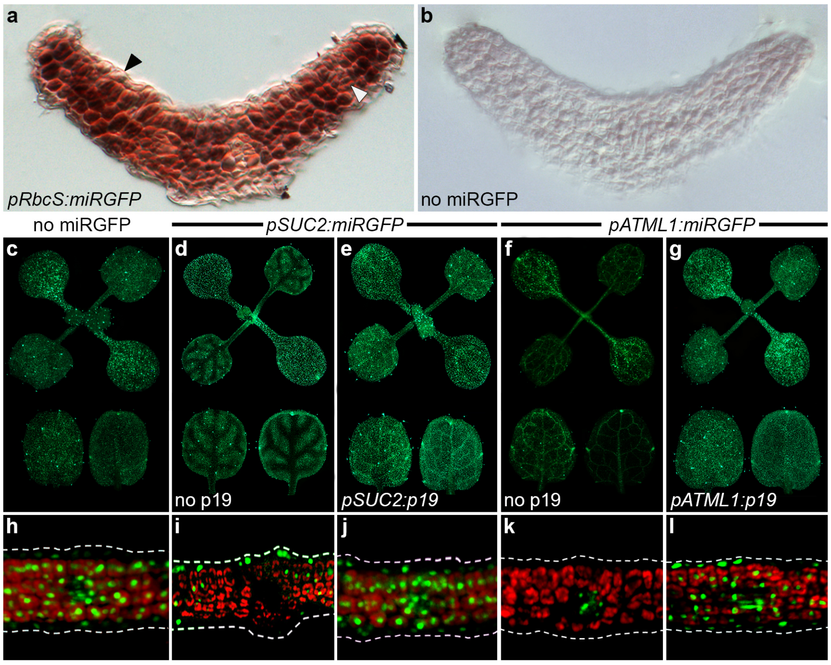

Supplementary Figure 4. miRGFP is the mobile silencing signal. **(a, b)** Small RNA *in situ* hybridization shows that **(a)** miRGFP produced in the mesophyll accumulates in the epidermis and vasculature (arrowheads) of *pRbcS:miRGFP* leaf primordia, and is **(b)** undetected in *p35S:3xNLS-GFP* primordia not expressing miRGFP (no miRGFP). **(c-i)** Compared to the uniform pattern of GFP fluorescence in **(c, h)** leaves of *p35S:3xNLS-GFP* (no miRGFP) seedlings, **(d, i)** *pSUC2:miRGFP* lines expressing miRGFP in the phloem companion cells show a non-cell autonomous pattern of GFP silencing around major veins that is (**e, j**) suppressed upon co-expression of the viral suppressor protein p19. Likewise, the non-cell autonomous pattern of GFP silencing seen in **(f, k)** *pATML1:miRGFP* lines expressing miRGFP in the epidermis is (**g, l**) suppressed upon co-expression of p19. **(c-g)** Fluorescence images of whole seedlings (top) and individual leaves (bottom); adaxial side, left; abaxial side, right. **(h-l)** Confocal microscopy images of transverse leaf sections.

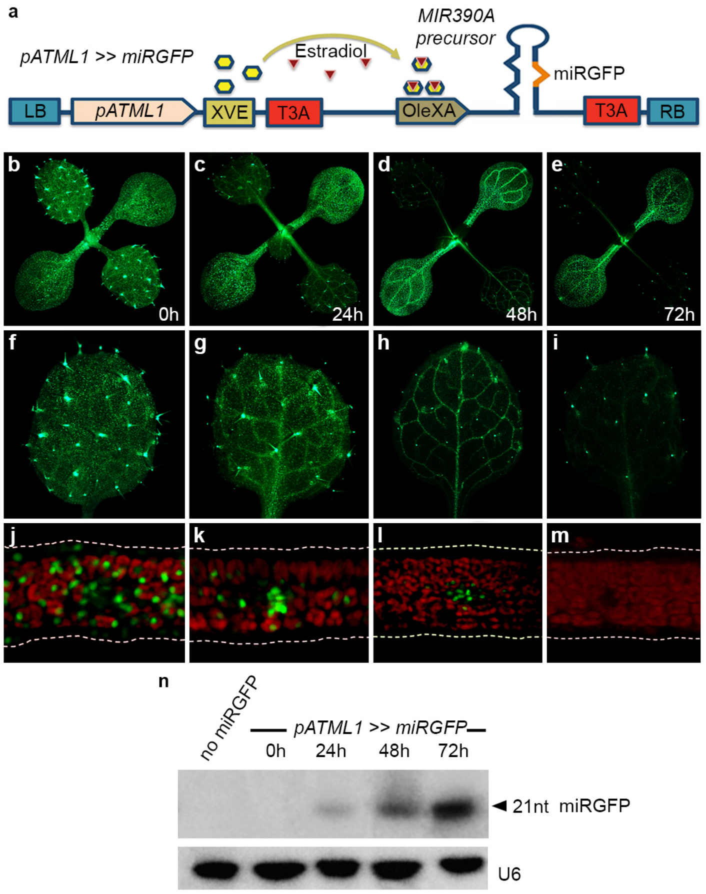

Supplementary Figure 5 The extent of miRGFP-directed GFP silencing correlates with miRNA levels at the source. **(a)** Schematic of the epidermal-specific, estradiol-inducible *pATML1>>miRGFP* construct. **(b-m)** Imaging of **(b-e)** whole seedlings, **(f-i)** mature leaves, and **(j-m)** transverse leaf sections shows that the miRGFP-directed silencing of GFP extends progressively from the epidermis into the mesophyll and vasculature of *pATML1>>miRGFP* seedlings upon induction with 20 μM estradiol for **(b, f, j)** 0, **(c, g, k)** 24, **(d, h, l)** 48, and **(e, i, m)** 72 hours. **(n)** Small RNA gel blot showing a time-dependent increase in miRGFP levels in *pATML1>>miRGFP* seedlings upon induction with 20 μM estradiol.

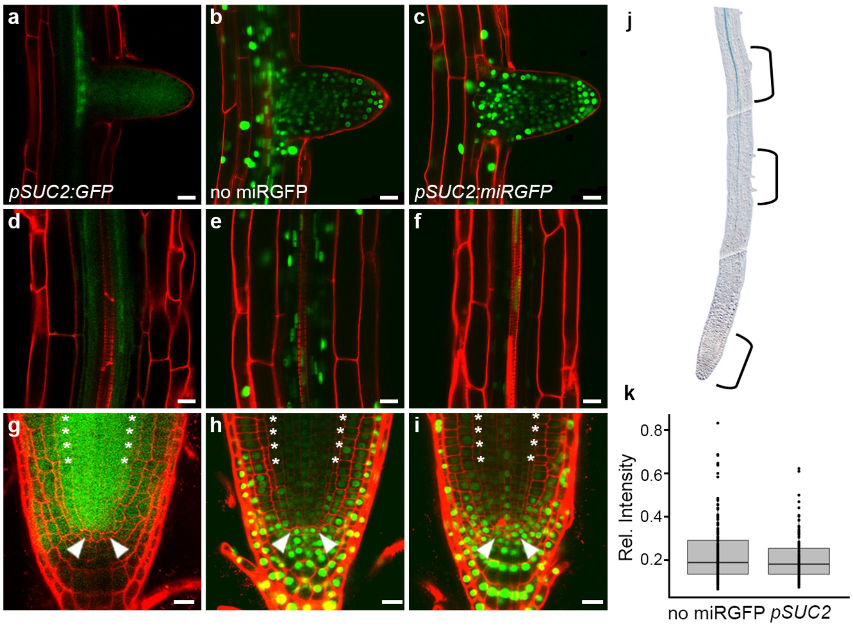

Supplementary Figure 6. miRNA mobility is regulated independently from small protein movement. **(a-i)** Optical confocal sections through **(a-c)** a lateral root meristem, **(d-f)** the root differentiation zone, and **(g-i)** the root apical meristem reveal opposite patterns of mobility for miRGFP and free GFP from phloem companion cells. **(a, d, g)** Free-GFP behaves **(d)** cell autonomously in the differentiation zone of *pSUC2:GFP* lines, but diffuses throughout the **(a)** lateral and **(g)** primary root meristem. Conversely, compared to the **(b, e, h)** ubiquitous pattern of GFP fluorescence in *p35S:3xNLS-GFP* (no miRGFP) roots, **(c, f, i)** *pSUC2:miRGFP* lines show a non-cell autonomous pattern of miRGFP-directed GFP silencing in **(f)** the root differentiation zone, and no silencing in **(c)** lateral or **(i)** primary root meristems. **(j)** GUS reporter activity shows the spatiotemporal pattern of *SUC2* promoter activity in root phloem companion cells. Box areas, regions imaged for the lateral root, differentiation zone, and root apical meristem. **(k)** Quantification of the mean GFP fluorescence intensity in endodermal cells ($n \geq 125$) normalised to fluorescence intensity in cells of the lateral root cap reveals this to not deviate significantly ($p>0.05$, two-sided Student's t test) in *p35S:3xNLS-GFP* (no miRGFP) versus *pSUC2:miRGFP* root meristems. Horizontal line, median; boxes, 1st and 3rd quartiles. Note, the same no miRGFP data is shown here and in Fig. 3c, as the *pSUC2:miRGFP* and *pRbcS:miRGFP* lines were analysed concurrently. Scale bars, 20 μm.

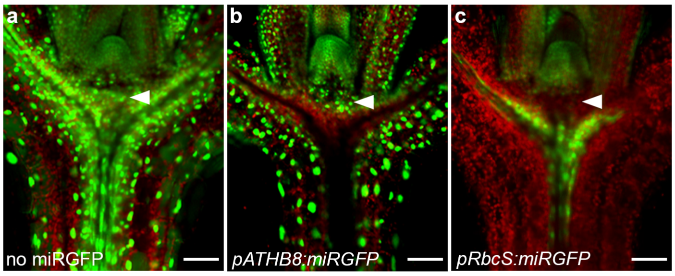

Supplementary Figure 7. miRNA mobility into the shoot stem cell niche is restricted. **(a-c)** Longitudinal sections through the shoot apex of **(a)** a *p35S:3xNLS-GFP* (no miRGFP) seedling and seedlings expressing miRGFP in **(b)** the procambium (*pATHB8:miRGFP*) and **(c)** the mesophyll (*pRbcS:miRGFP*) reveal a lack of miRGFP-directed GFP silencing in the shoot meristem of these lines. White arrowhead, pith region below the shoot apical meristem. Scale bars, 50 μm.

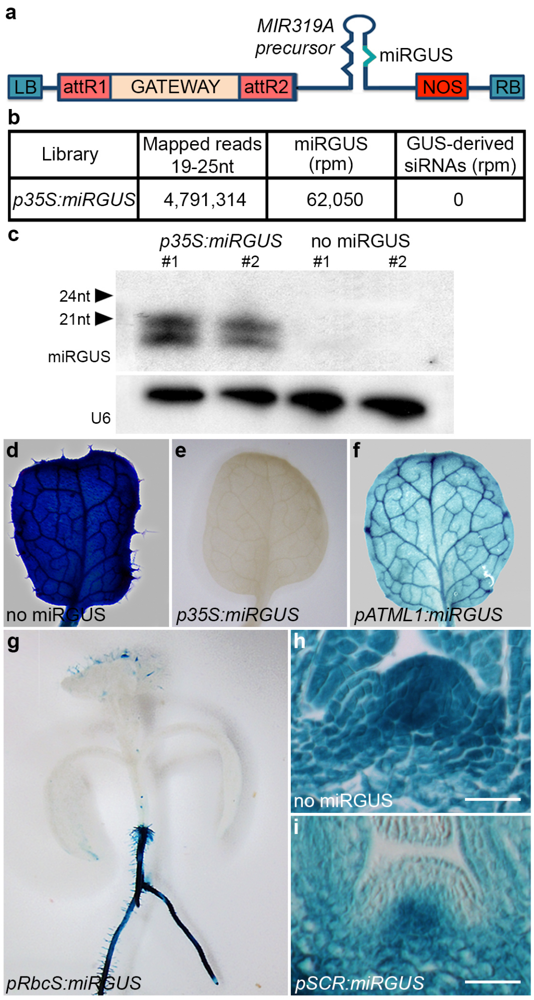

Supplementary Figure 8. miRGUS recapitulates the patterns of regulated miRGFP mobility. **(a)** Schematic of the Gateway-based miRGUS precursor construct. **(b)** Read counts for miRGUS and *GUS*-derived secondary siRNAs in reads per million (rpm) normalised to the total number of mapped 19-25 nt small RNA reads in libraries constructed from *p35S:miRGUS* seedlings. The absence of GUS-derived secondary siRNAs confirms the lack of transitivity in these lines. **(c)** Small RNA gel blot shows the miRGUS precursor in *p35S:miRGUS* lines generates both 20- and 21-nt miRNA species. U6 hybridization confirms near even loading of RNA samples. **(d, e)** GUS activity in **(d)** *p35S:GUS* (no miRGUS) seedlings is completely silenced in **(e)** lines ubiquitously expressing miRGUS (*p35S:miRGUS*). **(f)** A non-cell autonomous pattern of GUS silencing is detected in *pATML1:GUS* lines expressing miRGUS in the leaf epidermis. **(g)** miRGUS-directed GUS silencing in *pRbcS:miRGUS* seedlings is apparent in the hypocotyl and leaves but not in the root. **(h, i)** Longitudinal sections show GUS activity is detectable throughout **(h)** the *p35S:GUS* shoot apical meristem (no miRGUS), whereas a non-cell autonomous pattern of GUS silencing extends into the third meristem layer in **(i)** *pSCR:miRGUS* apices expressing miRGUS in the meristem tunica.

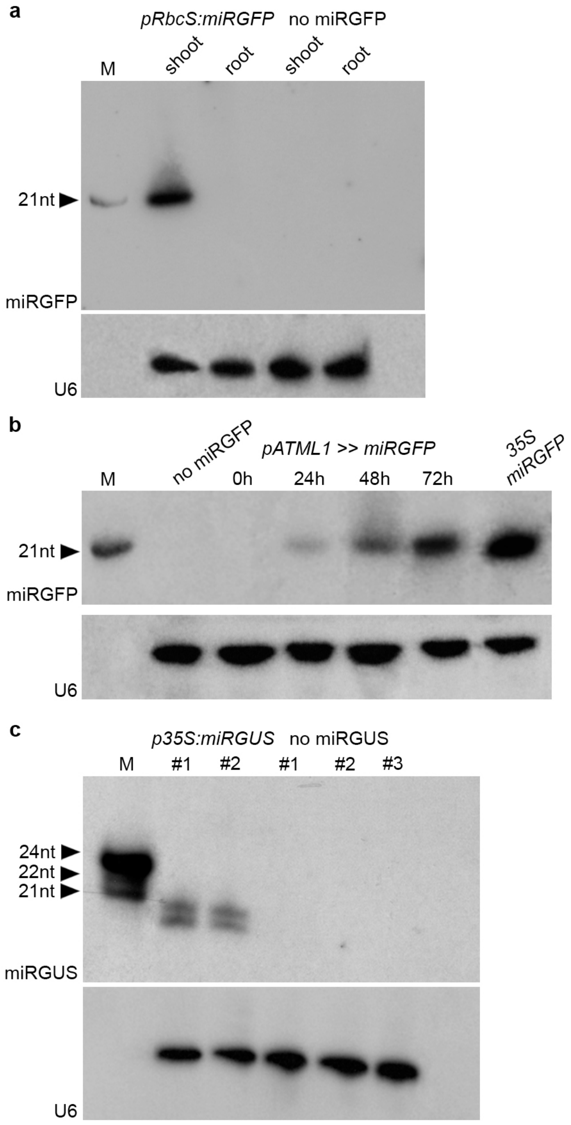

Supplementary Figure 9. Original small RNA northern blots. **(a)** Original images for the small RNA northern blots shown in Fig. 3d. **(b)** Original images for the small RNA northern blots shown in Supplementary Fig. 5n. **(c)** Original images for the small RNA northern blots shown in Supplementary Fig. 8b.

Supplementary Table 1. miRGFP read counts in libraries constructed from *pRbcS:miRGFP, pCLV3:miRGFP*, and *pSUC2:miRGFP* seedlings.

**Library**	**Mapped reads 19-25 nt**	**miRGFP (rpm)**	**GFP-derived siRNAs (rpm)**
pRbcS:miRGFP	14,985,989	42,250	0
pCLV3:miRGFP	27,294,977	14,808	0
pSUC2:miRGFP	15,817,134	30,117	0

Supplementary Table 2. Artificial small RNA and *in situ* LNA probe sequences

Artificial Small RNA	Sequence
miRGFP	TTGAAGTTCACCTTGATGCGG
miRGUS	TAATGAGTGACCGCATCGACC

Artificial Small RNA Precursor	Sequence*
MIR390A-based miRGFP precursor	GTAGAGAAGAATCTGTATGTA***TTGAAGTTCACCTTGATGCGG***ATGATGATCACATTCGTTATCTATTTTTT*CCGCATCAAGATGAACTTCAA*CATTGGCTCTTCTTACTAC
MIR319A-based miRGUS precursor	AG*GGCCGATGCGGTCTCTCATTT*TCACAGGTCGTGATATGATTCAATTAGCTTCCGACTCATTCATCCAAATACCGAGTCGCCAAAATTCAAACTAGACTCGTTAAATGAATGAATGATGCGGTAGACAAATTGGATCATTGATTCTCTTTGA***TAATGAGTGACCGCATCGACC***CT

LNA Probe	Sequence
asmiRGFP	CCGCATCAAGGTGAACTTCAA

* Mutated nucleotides are italicised and the mature miRNA is marked in bold

Supplementary Table 3. Cloning and qRT-PCR primers

Primers	Sequence
p2X35S-F	GGTACCGGTCTCAGAAGACCAGAGGGCTATTG
p2X35S-R	GAATTCGGTCCTCTCCAAATGAAATGAACTTCCTTATATAGAGG
pATML1-F	GAACTTACGTAGTTTACATGCATCTCATCCC
pATML1-R	AAACTCTATAGAACAGATCTCTTTTTTTTTTGAAG
pRbcS-F	TTTACCCTAACTACTCCTTTCTCAGTTGG
pRbcS-R	TATATAGTGGGAACCGCTAGAGGCAACTAGCCC
pMIR166A-F	TCGAGAATTACTATATCATAC
pMIR166A-R	TAGGGTTTCTGAATATATATC
pSUC2-F	AGTCATTATCAACTAGGGGTG
pSUC2-R	AAAGAAATTTCTTTGAGAGGG
pATHB8-F	AGTGTGCCTTATCACAGGGG
pATHB8-R	CTCTCTATTTAATTTTGTTCC
pSCR-F	GTAGGTACCACCACCACCGTCAACAATTTTGAATCC
pSCR-R	AGCTCGAGGGGGTTGGTCGTGAGATTGCATGG
pWUS-F	GTACTCATAGGAGTTTATAAATCAAAGGG
pWUS-R	TGTGTTTGATTCGACTTTTGTTCACAAAG
pCLV3-F	CGCGGTTTGTGTAAATGGTATTATTATC
pCLV3-R	CTACATGAACATAACACATGAATATTGAG
pWOX5-F	AGAACCTCGGGGATGAAGAC
pWOX5-R	AAACAGTTGAGGACTTTACATCTGAAC
P19-HA-F	AAACTAGTATGGAACGAGCTATACAAGG
P19-HA-R	AAGCGGCCGCGGTGATTTGCGGACTCTAGATTAAGCGTAGTCTGGG
pFK390-miRGFP-F	TTATAGGGGGGAAAAAAAGGTAG
pFK390-miRGFP-R	GAGACTAAAGATGAGATCTAATC
WOX5:GFP Gibson-1	GTCTTCATCCCCGAGGTTCTGCGGCCGCCTGCAGGTCGAC
WOX5:GFP Gibson-2	GCATGGTAGAACTATACAAATGATATCCCGCGGCCATGCTAGAGT
WOX5:GFP Gibson-3	AGCATGGCCGCGGGATATCATTTGTATAGTTCTACCATGCCA
WOX5:GFP Gibson-4	GTCGACCTGCAGGCGGCCGCAGAACCTCGGGGATGAAGAC
miRGFP stemloop	GTCGTATCCAGTGCAGGGTCCGAGGTATTCGCACTGGATACGACCGGCAT
miRGFP-F qRT-PCR	CCGCGTGGCTTGAAGTTCACCTTG
miRGFP-R qRT-PCR	CCAGTGCAGGGTCCGAGGTA
U6 stemloop	GTGCAGGGTCCGAGGTTTTGGACCATTTCTCGAT
U6-F	GGAACGATACAGAGAGAAGATTAGCA
U6-R	GTGCAGGGTCCGAGGT
